# Multi-beam reflections with flexible control of polarizations by using anisotropic metasurfaces

**DOI:** 10.1038/srep39390

**Published:** 2016-12-21

**Authors:** Hui Feng Ma, Yan Qing Liu, Kang Luan, Tie Jun Cui

**Affiliations:** 1 State Key Laboratory of Millimeter Waves, School of Information Science and Engineering, Southeast University, Nanjing 210096, China; 2 Synergetic Innovation Center of Wireless Communication Technology, Southeast University, Nanjing, 210096, China

## Abstract

We propose a method to convert linearly polarized incident electromagnetic waves fed by a single source into multi-beam reflections with independent control of polarizations based on anisotropic metasurface at microwave frequencies. The metasurface is composed of Jerusalem Cross structures and grounded plane spaced by a dielectric substrate. By designing the reflection-phase distributions of the anisotropic metasurface along the *x* and *y* directions, the *x*- and *y*-polarized incident waves can be manipulated independently to realize multi-beam reflections. When the *x*- and *y*-polarized reflected beams are designed to the same direction with equal amplitude, the polarization state of the beam will be only controlled by the phase difference between the *x*- and *y*-polarized reflected waves. Three examples are presented to show the multi-beam reflections with flexible control of polarizations by using anisotropic metasurfaces and excellent performance. Particularly, we designed, fabricated, and measured an anisotropic metasurface for two reflected beams with one linearly polarized and the other circularly polarized. The measurement results have good agreement with the simulations in a broad bandwidth.

Metasurface is two-dimensional (2D) planar surfaces constructed by a series of metallic or dielectric structures with subwavelength scale on the interface of two media with different refractive indices. The metallic structures can introduce discontinuous phase shifts on the surface, hence the electromagnetic waves (or lights) impinging on the metasurface will interact with the metallic structures to generate anomalous refractions and reflections, which obey the generalized Snell law^1^. Due to the scattering of metallic structures on the interface, the wavefronts of lights or waves can be modified to realize the desired functional devices in the regions of optics^2^,^3^,^4^,^5^,^6^,^7^,^8^,^9^, terahertz^10^,^11^,^12^,^13^,^14^, microwave^15^,^16^,^17^,^18^,^19^,^20^, and acoustics^21^,^22^,^23^,^24^,^25^. Otherwise, a grating metasuface also can be designed to capture light efficiently into surface plasmons by using transformation optics^26^. The polarization plays an important role in manipulating the lights or electromagnetic waves, realizing polarization beam splitters^9^,^18^, quarter-wave plates^7^, polarization-controlled plasmonic couplers^2^, and polarization converters^19^. Many efforts have been made to manipulate the polarizations by using metamaterials^27^,^28^,^29^ and metasurfaces^30^,^31^,^32^,^33^,^34^,^35^,^36^,^37^,^38^. However, these polarization modulations are mostly concentrated on a single radiation beam.

In this work, we propose a method to realize multi-beam reflections with independent control of polarizations based on anisotropic metasurfaces. The metasurfaces are composed of Jerusalem Cross structures and a grounded plane spaced by a dielectric substrate. A Ku-band coax-to-waveguide device is placed in front of the metasurfaces used as the feeding source, which can generate quasi-spherical incident waves with linear polarization^39^. The quasi-spherical incident waves can be reflected by the metasurfaces with high efficiency, while the reflected phases of *x*- and *y*-polarized waves can be controlled independently by changing the dimensions of the I-shaped structures on *x* and *y* directions, respectively. Based on the compensation method of geometrical optics and superposition of aperture fields, the *x*- and *y*-polarized reflected waves can be converted into multi-beam reflections independently with high directivities. In particular, when the *x*- and *y*-polarized waves are reflected to the same direction with equal amplitude, the polarization state of each reflected beam can be controlled independently by the phase difference between the *x*- and *y*-polarized reflected waves. Three full-wave simulation examples with single, dual and six reflected beams manipulated by anisotropic metasurfaces for both *x*- and *y*-polarized waves are provided to verify the proposed method, and the simulation results show good abilities of the designed metasurfaces in controlling the multi-beam reflections with independent control of polarization. For experimental verification, we design, fabricate and measure a metasurface, which can convert the incident quasi-spherical waves into two reflected beams with one linearly polarized and the other circularly polarized. The measured results demonstrate good performance of the designed metasurface, which has good agreement with the simulation.

## Results

### Theory and simulations

**Figure 1:**
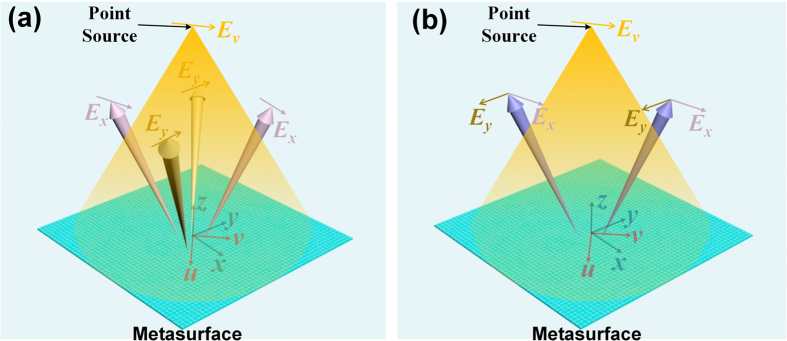
The sketch of the multi-beam reflection with independent control of *x*- and *y*-polarized waves fed by a linearly polarized point source with *v* polarization in front of the anisotropic metasurfaces. (**a**) The decomposed *x*- and *y*-polarized waves are reflected to the different directions. (**b**) The decomposed *x*- and *y*-polarized waves are reflected to the same directions.

The model of the proposed anisotropic metasurface fed by a point source is illustrated in Fig. 1. We assume that the point source is located at the top of the metasurface with coordinates of (0, 0, *R*_0_), and the coordinate origin is defined in the geometrical centre of the metasurface, as shown in Fig. 1(a). Then we divide the metasurface into *N* × *N* pixels, in which one pixel is a unit cell of the metasurface. The reflected phase of each pixel can be designed by changing the dimensions of the unit cell. To focus the reflected waves into plane waves with a designed deflection angle, the required reflected phase of the pixel at the position of (*x*_*i*_, *y*_*i*_, 0) can be calculated according to the phase compensation of geometrical optics


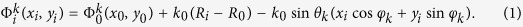


in which Φ_0_(*x*_0_, *y*_0_) is the reflection phase at the origin of the coordinate, *R*_0_ and *R*_*i*_ are the distances between the feeding source and pixels of (0, 0, 0) and (*x*_*i*_, *y*_*i*_, 0), respectively, and (*θ*_*k*_, *φ*_*k*_) is the deflection angle of the reflected plane waves.

Hence, if we design an anisotropic metasurface, whose phase distributions in both *x* and *y* directions are designed independently according to Eq. (1), then the *x*- and *y*-polarized waves can be controlled and reflected independently to the directions of (*θ*_*x*_, *φ*_*x*_) and (*θ*_*y*_, *φ*_*y*_), respectively. Furthermore, both *x*- and *y*-polarized waves can also be designed to multi-beam reflections by using superposition of the aperture fields^40^,^41^. To generate M_1_ and M_2_ (M_1_ and M_2_ are positive real numbers) reflected beams for the *x*- and *y*-polarized waves, the reflection phases of the metasurface at position of (*x*_*i*_, *y*_*i*_, 0) for *E*_*x*_ and *E*_*y*_ components can be calculated as^41^


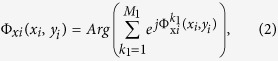



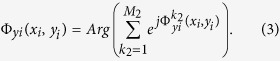


where 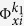 and 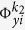 are the required phases at position of (*x*_*i*_, *y*_*i*_, 0) for the *k*_1th_ and *k*_2th_ reflected beams of the *x*- and *y*-polarized waves with the radiation directions of 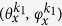 and 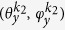, respectively, which can be obtained from Eq. (1). We further define a new rectangular coordinate *uvw* by rotating original rectangular coordinate *xyz* 45° clockwise along the *z* axis, as shown in Fig. 1. Hence, when the incident waves are fixed by *v* polarization, then the total electric field of *E*_*v*_ can be decomposed to *E*_*x*_ and *E*_*y*_ with |*E*_*x*_| = |*E*_*y*_|, and then the *x*- and *y*-polarized waves can be controlled and reflected independently to the different directions as demonstrated in Fig. 1(a) or the same directions as demonstrated in Fig. 1(b). Particularly, when the *x*- and *y*-polarized waves are reflected to the same directions, the polarization state of each beam can be only controlled independently by the phase difference of 
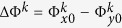
 between *x*- and *y*-polarized reflected waves according to Eq. (1), where 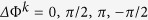 correspond to co-polarization, right-handed circular polarization, cross-polarization and left-handed circular polarization, respectively.

**Figure 2:**
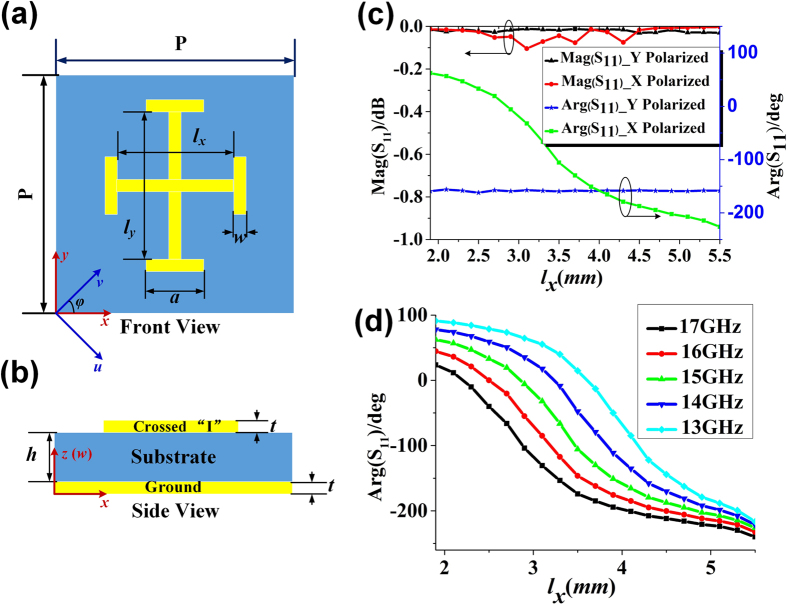
The unit cell of the anisotropic metasurface, and its amplitude and phase responses. (**a**) The front view of the unit cell composed of two orthogonal I-shaped structures in *x* and *y* directions. The new coordinate system *uvw* is defined by rotating coordinate system *xyz* 45° clockwise along the *z* axis. (**b**) The side view of the unit cell, the Jerusalem Cross structure and grounded plane is spaced by a dielectric substrate. (**c**) The amplitude and phase responses of the unit cell under *x*- and *y*-polarized incident waves, respectively, by only changing the length of *l*_*x*_. (**d**) The phase responses of the *x*-polarized reflected waves from 13 GHz to 17 GHz by changing the length of *l*_*x*_.

The metasurface, which is composed of a series of Jerusalem Cross structures and a grounded plane spaced by a dielectric substrate, has been proposed to realize above functions as shown in Fig. 2(a,b), in which the parameters are P = 6 mm, *a* = 1.8 mm, *h *= 2 mm, *w *= 0.2 mm, *t *= 0.018 mm and variables of *l*_*x*_ and *l*_*y*_. The dielectric substrate is F4B with relative permittivity of 2.65 and tangent loss of 0.001. The incident waves are almost totally reflected by the metasurface at 15 GHz, but the reflected phases of *E*_*x*_ (*E*_*y*_) can be manipulated from 70° to −230° by changing the length of *l*_*x*_ (*l*_*y*_) independently, which will not affect the reflection phase of *E*_*y*_ (*E*_*x*_) as shown in Fig. 2(c). The similar phase responses also can be achieved as the frequency changes from 13 GHz to 17 GHz as shown in Fig. 2(d). If a feeding source is placed in front of the metasurface with *v* polarization, then the incident waves are *v*-polarized waves, and the electric-field vector of ***E***_***v***_ can be decomposed to ***E***_***x***_ and ***E***_***y***_ with |***E***_***x***_| = |***E***_***y***_|, so the *x*- and *y*-polarized waves can be controlled independently by phase distributions of the metasurface in *x* and *y* directions, respectively. We remark that the feeding source used in our simulations and experiments is a linearly polarized Ku-band (12 GHz–18 GHz) coax-to-waveguide device with nearly equal E- and H-plane radiation patterns^39^. In the following, three specific examples are investigated by simulations to show the powerful ability of the proposed anisotropic metasurfaces in multi-beam reflections with independent control of polarizations.

We first consider that only one beam of both *x*- and *y*-polarized waves are reflected independently by the metasurface, whose phase distributions can be calculated from Eqs (2) and (3) by choosing M_1_ = M_2_ = 1 with the reflection directions of (*θ*_*x*_, *φ*_*x*_) and (*θ*_*y*_, *φ*_*y*_), respectively.


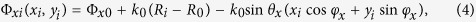



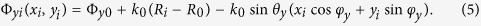


In which the Φ_*x*0_ and Φ_*y*0_ are the reflection phases at the origin of coordinate for *x*- and *y*-polarized waves, respectively, which can be the same or different.

**Figure 3:**
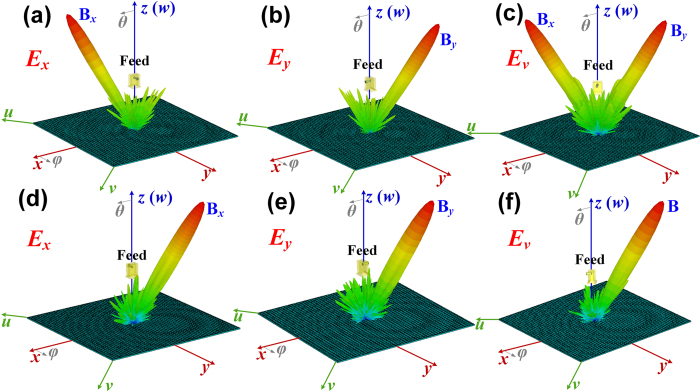
One reflected *x*- and *y*-polarized beam manipulated independently by the metasurfaces. (**a**–**c**) The *x*- and *y*-polarized waves are reflected to the different directions by the metasurface I: (**a**) the *x*-polarized waves are reflected to B_*x*_ (30°, 315°), (**b**) the *y*-polarized waves are reflected to B_*y*_ (30°, 135°), (**c**) the *x*- and *y*-polarized waves are separated and reflected to B_*x*_ (30°, 315°) and B_*y*_ (30°, 135°), respectively, for *v*-polarized incident waves. (**d**–**f**) The *x*- and *y*-polarized beams are reflected to the same direction by the metasurface II: (**d**) the *x*-polarized waves are reflected to B_*x*_ (30°, 135°), (**e**) the *y*-polarized waves are also reflected to B_*y*_ (30°, 135°), (**f**) the *x*- and *y*-polarized waves are reflected to the same direction of B (30°, 135°) for *v*-polarized incident waves.

**Figure 4:**
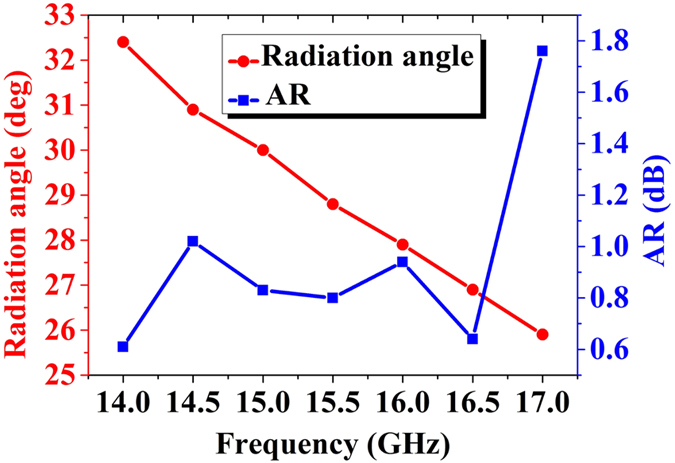
The simulated beam deflections and axis ratios of the reflected beam shown in Fig. 3(f) from 14 GHz to 17 GHz.

The metasurface I is designed to reflect the *x*- and *y*-polarized waves to the different directions of (*θ*_*x*_ = 30°, *φ*_*x*_ = 315°) and (*θ*_*y*_ = 30°, *φ*_*y*_ = 135°) at 15 GHz as shown in Fig. 3(a,b), respectively. When the incident waves are fixed as *v* polarization, the *x*- and *y*-polarized waves can be separated and reflected independently to the directions of (30°, 315°) and (30°, 135°), respectively, as shown in Fig. 3(c). The metasurface II is designed to reflect the *x*- and *y*-polarized waves to the same direction of (*θ*_*x*_ = *θ*_*y*_ = 30°, *φ*_*x*_ = *φ*_*y*_ = 135°) at 15 GHz as shown in Fig. 3(e,f). When the incident waves are fixed as *v* polarization, both *x*- and *y*-polarized waves are reflected to the same direction of (30°, 135°) and combined to a single beam B, as illustrated in Fig. 3(f), and the polarization state of the beam can be controlled by ΔΦ = Φ_*x*0_ − Φ_*y*0_. Here, we design ΔΦ = 90° to generate a circularly polarized beam, whose axial ratios are smaller than 1.8 and the radiation angle of *θ* steers from 32.5° to 25.8° from 14 GHz to 17 GHz, as shown in Fig. 4.

We further consider that two beams of both *x*- and *y*-polarized waves are reflected independently by the metasurface, whose phase distributions can be calculated from Eqs (2) and (3) by choosing M_1_ = M_2_ = 2 respectively.


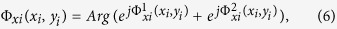



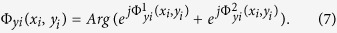


In which


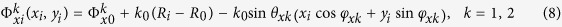



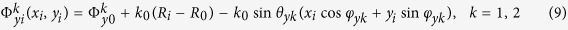


where the 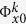 and 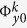 are the reflection phases at the origin of coordinate for the k_th_ reflected beams of the *x*- and *y*-polarized waves, respectively, which can be the same or different.

**Figure 5:**
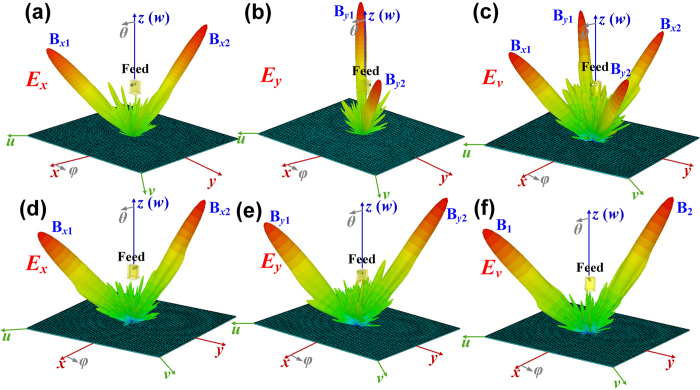
Two reflected *x*- and *y*-polarized beams manipulated by the metasurfaces. (**a**–**c**) The *x*- and *y*-polarized waves are reflected to the different directions by the metasurface III: (**a**) the *x*-polarized waves are reflected to B_*x*1_ (30°, 315°) and B_*x*2_ (30°, 135°), (**b**) the *y*-polarized waves are reflected to B_*y*1_ (30°, 45°) and B_*y*2_ (30°, 225°), (**c**) the *x*-polarized waves are reflected to B_*x*1_ (30°, 315°) and B_*x*2_ (30°, 135°) and the *y*-polarized waves are reflected to B_*y*1_ (30°, 45°) and B_*y*2_ (30°, 225°) at the same time for *v*-polarized incident waves. (**d**–**f**) The *x*- and *y*-polarized waves are reflected to the same directions by the metasurface IV: (**d**) the *x*-polarized waves are reflected to B_*x*1_ (30°, 315°) and B_*x*2_ (30°, 135°), (**e**) the *y*-polarized waves are also reflected to B_*y*1_ (30°, 315°) and B_*y*2_ (30°, 135°), (**f**) the *x*- and *y*-polarized waves are reflected to the same directions of B_1_ (30°, 315°) and B_2_ (30°, 135°) at the same time for *v*-polarized incident waves.

**Figure 6:**
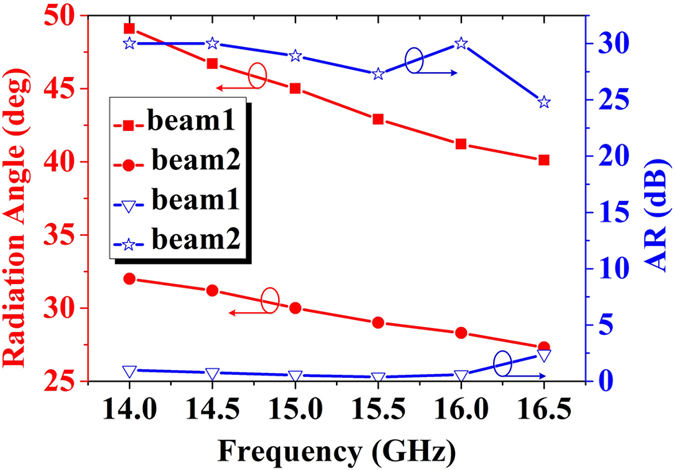
The simulated beam deflections and axis ratios of the B1 and B2 shown in Fig. 5(f) from 14 GHz to 16.5 GHz.

The metasurface III is designed to separate and reflect *x*-polarized waves to the directions of B_*x*1_ (*θ*_*x*1_ = 45°, *φ*_*x*1_ = 315°) and B_*x*2_ (*θ*_*x*2_ = 30°, *φ*_*x*2_ = 135°) as shown in Fig. 5(a), and the *y*-polarized waves to the directions of B_*y*1_ (*θ*_*y*1_ = 45°, *φ*_*y*1_ = 225°) and B_*y*2_ (*θ*_*y*2_ = 30°, *φ*_*y*2_ = 45°) as shown in Fig. 5(b). When the incident waves are fixed as *v* polarization, the *x*- and *y*-polarized waves can be separated and reflected independently to the different directions at same time, respectively, as shown in Fig. 5(c). The metasurface IV is also designed to separate both *x*- and *y*-polarized waves to two beams but deflected to the same directions of B_1_ (*θ*_1_ = 45°, *φ*_1_ = 315°) and B_2_ (*θ*_2_ = 30°, *φ*_2_ = 135°) as shown in Fig. 5(d,e), respectively. When the incident waves are fixed as *v* polarization, the *x*- and *y*-polarized waves also can be separated and reflected to two beams, but deflect to the same directions as shown Fig. 5(f). The polarization states of B_1_ and B_2_ can be manipulated independently by the phase differences between the *x*- and *y*-polarized waves for each beam, respectively. Here, we design 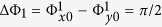 and 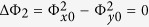 to realize circular and linear polarizations for B1 and B2, respectively. The simulated results show that the B1 and B2 are directed to 45° and 30° at 15 GHz, which are decreased from 49° to 41° and 32° to 28° as frequency increases from 14 GHz to 16.5 GHz, respectively, as red solid lines shown in Fig. 6. The axial ratios of B1 are lower than 2.5dB in whole frequency band to exhibit a good characteristic of circular polarization, and the axial ratios of B2 are larger than 25dB in whole frequency band to exhibit a good characteristic of linear polarization, as blue solid lines shown in Fig. 6.

**Figure 7:**
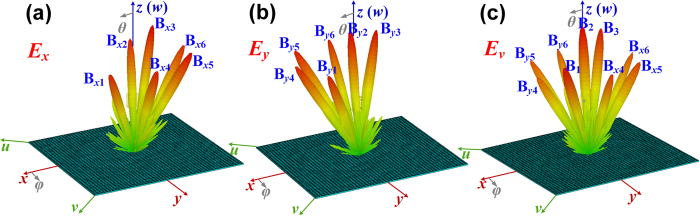
Six reflected *x*- and *y*-polarized beams manipulated by metasurfaces. (**a**) The *x*-polarized waves are separated and reflected to six different directions. (**b**) The *y*-polarized waves are separated and reflected to six different directions. (**c**) The *x*- and *y*-polarized waves are reflected to the six different directions at the same time for *v*-polarized incident waves, in which B_*x*1_, B_*x*2_ and B_*x*3_ are reflected to the same directions with B_*y*1_, B_*y*2_ and B_*y*3_ defined by B_1_, B_2_ and B_3_, respectively.

We finally design the metasurface V to reflect six beams of both *x*- and *y*-polarized waves, respectively. Similarly, the phase distributions of the metasurface also can be calculated from Eqs (2) and (3) by choosing M_1_ = M_2_ = 6. Figure 7(a) shows that the *x-*polarized incident waves are separated to six beams with the directions of B_*x*1_ (*θ*_*x*1_ = 30°, *φ*_1_ = 45°), B_*x*2_ (*θ*_*x*2_ = 0, *φ*_*x*2_ = 0), B_*x*3_ (*θ*_*x*3_ = 30°, *φ*_*x*3_ = 225°), B_*x*4_ (*θ*_*x*4_ = 30°, *φ*_*x*4_ = 90°), B_*x*5_ (*θ*_*x*5_ = 30°, *φ*_*x*5_ = 135°) and B_*x*6_ (*θ*_*x*6_ = 30°, *φ*_*x*6_ = 180°) after reflected by the metasurface. Figure 7(b) shows that the *y*-polarized incident waves are also separated to six radiation beams with the directions of B_*y*1_ (*θ*_*x*1_ = 30°, *φ*_*y*1_ = 45°), B_*y*2_ (*θ*_*x*2_ = 0, *φ*_*y*2_ = 0), B_*y*3_ (*θ*_*x*3_ = 30°, *φ*_*y*3_ = 225°), B_*y*4_ (*θ*_*x*4_ = 30°, *φ*_*y*4_ = 0), B_*y*5_ (*θ*_*x*5_ = 30°, *φ*_*y*5_ = 315°) and B_*y*6_ (*θ*_*x*6_ = 30°, *φ*_*y*6_ = 270°) after reflected by the metasurface. Hence, when the incident waves are fixed as *v* polarization, the *x*- and *y*-polarized waves can be manipulated independently by the metasurface at the same time to generate six reflected beams, respectively. However, because the B_*x*1_, B_*x*2_ and B_*x*3_ of *x*-polarized waves are deflected to the same directions with the B_*y*1_, B_*y*2_ and B_*y*3_ of *y*-polarized waves, and the beams are combined to B_1_, B_2_ and B_3_, respectively, as shown in Fig. 7(c), whose polarization states can be controlled independently by designing the phase differences of ΔΦ_1_, ΔΦ_2_ and ΔΦ_3_, respectively. Here, the ΔΦ_1_, ΔΦ_2_ and ΔΦ_3_ are designed to π/2, 0 and −π/2 to make B_1_, B_2_ and B_3_ right-handed circular polarization, linear polarization and left-handed circular polarization, respectively. The performance of each beam can be verified by calculating the axial ratios as above discussions, which are not provided here in consideration of the length of the paper.

### Experimental results

**Figure 8:**
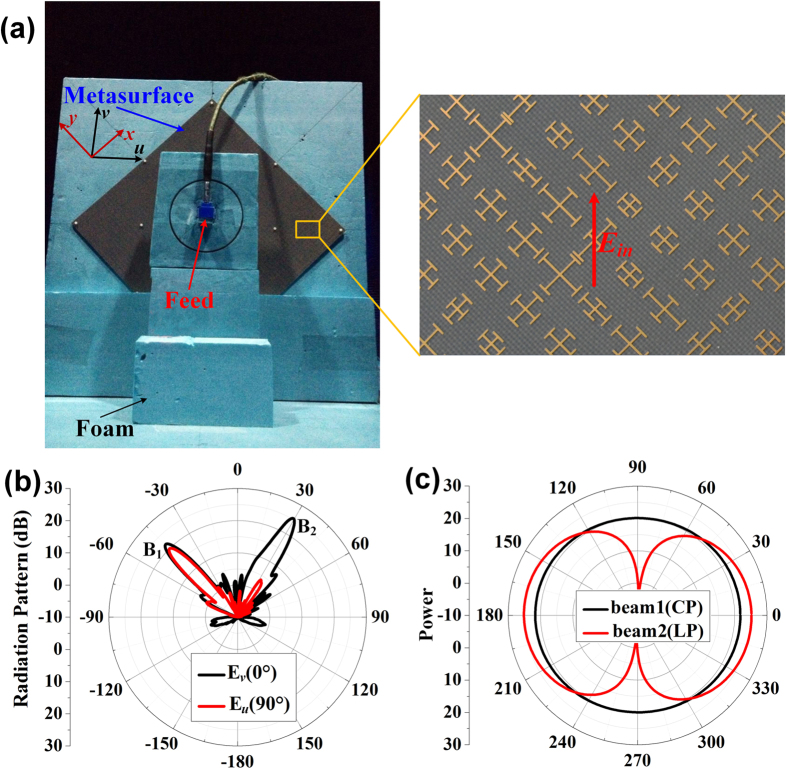
The experimental setup of metasurface IV and its measured results at 15 GHz. (**a**) The metasurface IV fed by a Ku-band rectangular waveguide. (**b**) The measured far-field radiation patterns on *uow* plane for *v* and *u* polarizations, respectively. (**c**) The measured power distributions of B_1_ and B_2_ at 15 GHz, respectively, by rotating the receiving linearly polarized antenna 360° in the plane paralleling to its aperture.

In experiments, we designed, fabricated and measured the metasurface IV, which can convert the incident *x*- and *y*-polarized waves into two reflected beams, respectively. The experimental sample is placed on a measurement platform, as demonstrated in Fig. 8(a), and the measured far-field patterns of *v*- and *u*-polarized reflected waves at 15 GHz are shown in Fig. 8(b). The measured results show that both *v*- and *u*-polarized waves are radiated to *θ*_1_ = −45° (beam 1) with nearly equal amplitude, but only the *v*-polarized waves are radiated to *θ*_2_ = 30° (beam 2). The measured amplitudes of both *v*- and *u*-polarized waves for beam 1 are about 3dB smaller than that of beam 2, which is because the beam 1 is designed to be a circular polarization with only half of the energy received by the linearly polarized receiving horn antenna in measurement. We also notice that the sidelobes are nearly 20dB smaller than the main beam of the beam 2, which is extremely low compared to the main beam. Hence, we can conclude that all the incident power is almost divided equally into the beam 1 and beam 2 with high radiation efficiency. In order to investigate the polarization of each beam, we measured the power distributions of *E* components for the beam 1 and beam 2 on the plane paralleling to their wavefronts, as shown by the black and red solid lines in Fig. 8(c), respectively. The measured results show good performances of the circular polarization for beam 1 and linear polarization for beam 2 at 15 GHz, respectively, which have a good agreement with the simulations, as shown in Fig. 5(f).

**Figure 9:**
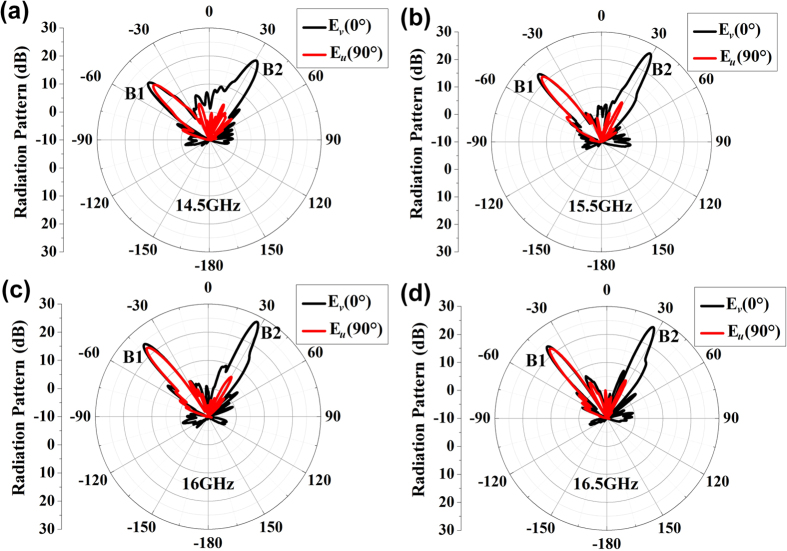
The measured far-field radiation patterns at different frequencies. (**a**) 14.5 GHz, (**b**) 15.5 GHz, (**c**) 16 GHz, (**d**) 16.5 GHz.

**Figure 10:**
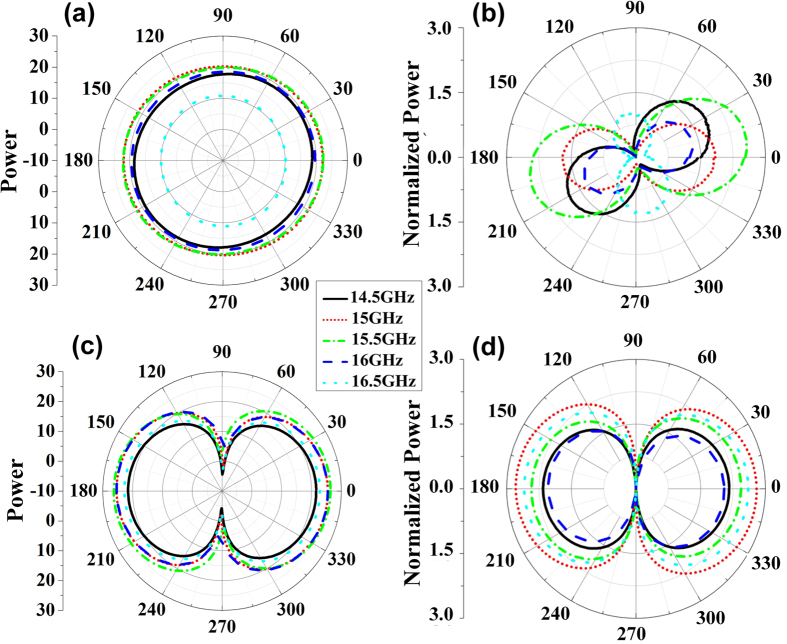
The measured power distributions of B_1_ and B_2_ at different frequencies. (**a**) The measured power distributions of B_1_, (**b**) the normalized power distributions of B_1_ calculated by δR = 20(log|*E|* − log|*E*_min_|), (**c**) the measured power distributions of B_2_, (**d**) the normalized power distributions of B_2_ calculated by δR = 20(log|*E|* − log|*E*_min_|).

The far-field patterns at 14.5 GHz, 15.5 GHz, 16 GHz and 16.5 GHz are also measured as shown in Fig. 9, which show good radiation performances except for slight deviations of beam 1 and beam 2 to the directions of −45° and 30°, respectively. Figure 10(a,c) are the measured power distributions of beam 1 and beam 2 in the plane paralleling to their wavefronts. Figure 10(b,d) are the normalized results of power distributions calculated by δR = 20(log|*E|*-log|*E*_min_|), in which AR = max(δR) is the axis ratio of each beam. The axis ratios of beam 1 are smaller than 3dB at all frequencies as shown in Fig. 10(b), which demonstrate good performances of circular polarizations, and the axis ratios of beam 2 are larger than 20dB at all frequencies as shown in Fig. 10(d), which demonstrate good performances of linear polarizations. Hence, the working bandwidth of the designed metasurface can cover from 14.5 GHz to 16.5 GHz. We notice that the measured power becomes smaller and smaller as the frequency deviates further and further away from 15 GHz as shown in Fig. 10(a,c), this is because the main beams deviate away from the measured angles of −45° and 30° at these frequencies for beam 1 and beam 2, respectively.

### Discussion

We have proposed a method based on the anisotropic metasurface to manipulate incoming quasi-spherical linearly polarized waves into multi-beam reflection with independent control of polarizations. The metasurface is composed of Jerusalem Cross structures and a grounded plane spaced by a dielectric substrate, which can control the *x*- and *y*-polarized reflected waves independently by designing the phase distributions in *x* and *y* directions, respectively. Particularly, when the *x*- and *y*-polarized reflected beams are reflected to the same direction, the polarization state of the beam can be controlled by designing the phase difference between the *x*- and *y*-polarized reflected waves. The full-wave simulated results verify that the reflected beams of *x*- and *y*-polarized waves can be manipulated independently, such as the number of beams, the reflection angles and polarization states. The metasurface, which can manipulate incoming quasi-spherical waves into two reflected beams with different polarizations, has been fabricated and measured, and the measured results have good agreement with the simulation. The proposed method in this paper provides a good choice for people to manipulate the reflected beams and their polarizations independently, which may have potential applications in satellite communications, millimetre wave image system, radar system and so on.

## Methods

To design the metasurface IV, the required phase distributions of the metasurface for both *E*_*x*_ and *E*_*y*_ components at 15 GHz were first calculated from Eqs (6)–(9), 13, 14, 15, in which the relative parameters are *θ*_*x*1_ = *θ*_*y*1_ = 45°, *φ*_*x*1_ = *φ*_*y*1_ = 315°, *θ*_*x*2_ = *θ*_*y*2_ = 30°, *φ*_*x*2_ = *φ*_*y*2_ = 135°, *R*_0_ = 91.8 mm, 
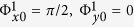
_and_ 
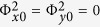
. Then, the Jerusalem Cross structure with different *l*_*x*_ and *l*_*y*_ was chosen to realized the required Φ_*x*i_ and Φ_*y*i_ in each pixel of the metasurface according to the relationship between the dimension of the Jerusalem Cross structure and the reflected phase as demonstrated in Fig. 2(c).

The dimension of the designed metasurface is 306 mm × 306 mm with 51 unit cells in both *x* and *y* directions, which is fabricated by using printed circuit board (PCB) of F4B with relative permittivity of 2.65 and loss tangent of 0.001. In measurement, a Ku-band coax-to-waveguide device is placed in front of the metasurface as the feeding source to generate *v*-polarized quasi-spherical incident waves, and the distance between the feeding source and centre of the metasurface is 91.8 mm. A linearly polarized receiving horn antenna is placed in the other side of the anechoic chamber to receive the far-field power of the reflected waves by rotating the sample 360° in horizontal plane, as shown in Fig. 8(b). In order to investigate the polarization of each beam, we first rotate the sample by −45° in the horizontal plane to make the beam 1 be directed to the receiving horn. Then, we rotate the receiving horn by 360° in the plane parallel to its aperture with interval of 1° to measure the power distribution of *E* component for the beam 1, as shown by the black solid line in Fig. 8(c). The similar method also has also been used to measure the power distribution of *E* component for the beam 2, as shown by the red solid line in Fig. 8(c). We remark that the initial polarization (0 degree) of the receiving horn is the *v* polarization.

## Additional Information

**How to cite this article**: Ma, H. F. *et al*. Multi-beam reflections with flexible control of polarizations by using anisotropic metasurfaces. *Sci. Rep.* **6**, 39390; doi: 10.1038/srep39390 (2016).

**Publisher's note:** Springer Nature remains neutral with regard to jurisdictional claims in published maps and institutional affiliations.
